# Genome Triplication Leads to Transcriptional Divergence of *FLOWERING LOCUS C* Genes During Vernalization in the Genus *Brassica*

**DOI:** 10.3389/fpls.2020.619417

**Published:** 2021-02-09

**Authors:** Ayasha Akter, Etsuko Itabashi, Tomohiro Kakizaki, Keiichi Okazaki, Elizabeth S. Dennis, Ryo Fujimoto

**Affiliations:** ^1^Graduate School of Agricultural Science, Kobe University, Kobe, Japan; ^2^Department of Horticulture, Faculty of Agriculture, Bangladesh Agricultural University, Mymensingh, Bangladesh; ^3^Institute of Vegetable and Floriculture Science, National Agriculture and Food Research Organization (NARO), Tsu, Japan; ^4^Graduate School of Science and Technology, Niigata University, Niigata, Japan; ^5^CSIRO Agriculture and Food, Canberra, ACT, Australia; ^6^School of Life Sciences, Faculty of Science, University of Technology, Sydney, Broadway, NSW, Australia

**Keywords:** *FLOWERING LOCUS C*, vernalization, epigenetics, non-coding RNA, *Brassica*

## Abstract

The genus *Brassica* includes oil crops, vegetables, condiments, fodder crops, and ornamental plants. *Brassica* species underwent a whole genome triplication event after speciation between ancestral species of *Brassica* and closely related genera including *Arabidopsis thaliana*. Diploid species such as *Brassica rapa* and *Brassica oleracea* have three copies of genes orthologous to each *A. thaliana* gene, although deletion in one or two of the three homologs has occurred in some genes. The floral transition is one of the crucial events in a plant’s life history, and time of flowering is an important agricultural trait. There is a variation in flowering time within species of the genus *Brassica*, and this variation is largely dependent on a difference in vernalization requirements. In *Brassica*, like in *A. thaliana*, the key gene of vernalization is *FLOWERING LOCUS C* (*FLC*). In *Brassica* species, the vernalization response including the repression of *FLC* expression by cold treatment and the enrichment of the repressive histone modification tri-methylated histone H3 lysine 27 (H3K27me3) at the *FLC* locus is similar to *A. thaliana*. *B. rapa* and *B. oleracea* each have four paralogs of *FLC*, and the allotetraploid species, *Brassica napus*, has nine paralogs. The increased number of paralogs makes the role of *FLC* in vernalization more complicated; in a single plant, paralogs vary in the expression level of *FLC* before and after vernalization. There is also variation in *FLC* expression levels between accessions. In this review, we focus on the regulatory circuits of the vernalization response of *FLC* expression in the genus *Brassica*.

## Introduction

*Brassica* is an economically important genus including many agricultural crops such as Chinese cabbage and turnip (*Brassica rapa* L.), cabbage, broccoli, cauliflower, kale, and kohlrabi (*Brassica oleracea* L.), and oilseed or rapeseed and rutabaga (*Brassica napus* L.). Three diploid species, *B. rapa*, *Brassica nigra* L., and *B. oleracea* are denoted as the A, B, and C genomes, respectively. Allotetraploid species, *Brassica juncea* L. (AABB), *Brassica carinata* L. (BBCC), and *B. napus* (AACC) contain two diploid genomes, and this genomic relationship is known as the “Triangle of U” (Nagaharu, 1935). *Arabidopsis thaliana* L. is a close relative species to the genus *Brassica*, and both are contained in the Brassicaceae.

The genus *Brassica* needs vernalization for induction of flowering except for early flowering accessions, which have lost the genes responsible for a vernalization requirement. The length of cold treatment required for a vernalization response varies between accessions within a species; for example, the natural variation in flowering time in *B. napus* in response to vernalization is characterized into three groups: winter type (high vernalization requirement), semiwinter type (intermediate vernalization requirement), and spring type (low or no vernalization requirement) (Raman et al., 2016).

The molecular mechanism of vernalization is well studied in *A. thaliana*, and genes involved in vernalization networks have been characterized (Berry and Dean, 2015; Whittaker and Dean, 2017). No one doubts that *FLOWERING LOCUS C* (*FLC*) is a key gene in vernalization. *FLC* encodes a MADS-box transcription factor and acts as a floral repressor (Michaels and Amasino, 1999; Sheldon et al., 1999). *FLC* is expressed before cold exposure, and FRIGIDA (FRI) is involved in activation of *FLC* expression. *FLC* expression is repressed during cold exposure through epigenetic regulation (Groszmann et al., 2011; Berry and Dean, 2015; Whittaker and Dean, 2017). Upon return to warm conditions, silencing of *FLC* is maintained (Berry and Dean, 2015; Whittaker and Dean, 2017).

In the genus *Brassica*, *FLC* is also a key gene in the vernalization process, and the mechanism of vernalization in the genus *Brassica* has much in common with that in *A. thaliana* (Itabashi et al., 2018). However, molecular mechanisms specific to *Brassica* have also been identified. In this review, we describe the research findings on vernalization in the genus *Brassica*.

## Multiple Copies of *FLC* Paralogs Are Generated by Whole Genome Triplication or Allotetrapolyploidization in the Genus *Brassica*

The tribe Brassiceae has undergone a whole genome triplication after speciation, and the whole genome sequence of *B. rapa* confirmed the triplication of the *B. rapa* genome relative to *A. thaliana* (Wang et al., 2011). The total number of genes in *B. rapa* is much less than three times the gene number in *A. thaliana* due to gene loss after the whole genome triplication in *B. rapa* (Wang et al., 2011). In the genus *Brassica*, three subgenomes, the least fractioned subgenome (LF) and two more fractionated subgenomes (MF1 and MF2), are recognized. In *B. rapa*, the LF subgenome retains 70% of *A. thaliana* orthologs, while 46 and 36% of *A. thaliana* orthologs are retained in MF1 and MF2 subgenomes, respectively (Wang et al., 2011).

*B. rapa* has multiple copies of orthologs for each *A. thaliana* gene. For example, there are two *FRI* paralogs (*BrFRIa*, *BrFRIb*) and four *FLC* paralogs (*BrFLC1*, *BrFLC2*, *BrFLC3*, *BrFLC5*) (Table 1; Schranz et al., 2002; Wang et al., 2017; Shea et al., 2018). *BrFLC1*, *BrFLC2*, and *BrFLC3* are located on LF, MF2, and MF1 subgenomes, respectively, indicating that whole genome triplication state is preserved. However, it is not clear how *BrFLC5* was generated (Wang et al., 2011). In *B. oleracea*, there are two *FRI* and four *FLC* paralogs (Table 1), and *BoFLC5* is a pseudogene (Schranz et al., 2002; Irwin et al., 2012; Itabashi et al., 2019). The allotetraploid species, *B. napus*, has four *FRI* and nine *FLC* paralogs (Table 1; Chalhoub et al., 2014; Shea et al., 2018; Yi et al., 2018; Schiessl et al., 2019). The relationship of orthologs in *FRI* and *FLC* among three species is shown in Table 1.

**TABLE 1:** *FRI* and *FLC* paralogs in three species of the genus *Brassica*.

	*Brassica rapa* (AA)	var. *pekinensis* line Chiifu-401-42		*Brassica oleracea* (CC)	var. *capitata* line 02-12	*Brassica napus*	European winter oilseed cultivar “Darmor-bzh”
		ver. 1.5	ver. 3.0				
*FLC1*	*BrFLC1* (A10)	Bra009055	BraA10g027720.3C			*BnaFLC.A10*	BnaA10g22080D
				*BoFLC1* (C09)	Bol043693	*BnaFLC.C09A*	BnaC09g46500D
						*BnaFLC.C09B*	BnaC09g46540D
*FLC2*	*BrFLC2* (A02)	Bra028599	BraA02g003340.3C			*BnaFLC.A02*	BnaA02g00370D
				*BoFLC2* (C02)		*BnaFLC.C02*	BnaC02g00490D
*FLC3*	*BrFLC3* (A03)	Bra006051	BraA03g004170.3C			*BnaFLC.A03A*	BnaA03g02820D
				*BoFLC3* (C03)	Bol008758	*BnaFLC.C03A*	BnaC03g04170D
*FLC5*	*BrFLC5* (A03)	Bra022771	BraA03g015950.3C			*BnaFLC.A03B*	BnaA03g13630D
				*BoFLC5* (C03)		*BnaFLC.C03B*	BnaC03g16530D
*FRIa*	*BrFRIa* (A03)	Bra029192	BraA03g015670.3C			*BnaFRI.A03*	BnaA03g13320D
				*BoFRIa* (C03)	Bol028107	*Bna.FRI.C03*	BnaC03g16130D
*FRIb*	*BrFRIb* (A10)	Bra035723	BraA10g009310.3C			*BnaFRI.A10*	BnaA10g05850D
				*BoFRIb* (C09)	Bol004294	*Bna.FRI.C09*	BnaC09g27290D

Multiple copies of orthologs may lead to subfunctionalization in the genus *Brassica*. In *A. thaliana*, loss of function of the single-copy *AtFRI* is the main cause of natural variation in flowering time (Berry and Dean, 2015; Whittaker and Dean, 2017). However, there are few reports of *FRI* being a major contributor of flowering time variation in the genus *Brassica*. Since both FRIa and FRIb act as activators of *FLC* in the genus *Brassica* (Irwin et al., 2012; Takada et al., 2019), they complement each other so that there is little chance of simultaneous loss of FRI function in nature. In contrast, there are reports that loss of *FLC* function results in variation in flowering time in the genus *Brassica*.

## Quantitative Trait Locus Analysis Shows That FLC Is a Flowering Time Regulator

To identify the key genes involved in vernalization, quantitative trait locus (QTL) analyses have been performed. QTLs affecting flowering time have been identified in different populations in *B. rapa*, *B. oleracea*, and *B. napus* (Shea et al., 2018). At the beginning of QTL analyses in the genus *Brassica*, a population derived from early and late flowering parental accessions was used. Some QTLs overlapped with the region covering the *FLC* gene, and some indicated that loss of FLC function results in early flowering (Kole et al., 2001; Schranz et al., 2002; Lou et al., 2007; Okazaki et al., 2007; Li et al., 2009; Zhao et al., 2010); loss of *FLC2* function has been detected in early flowering accessions of *B. rapa* and *B. oleracea* (Table 2; Okazaki et al., 2007; Li et al., 2009; Wu et al., 2012). In cauliflower (*B. oleracea*), there is an association between *BoFLC2* allelic variation and floral induction within populations of inbred lines (Ridge et al., 2015).

**TABLE 2:** Example of how variation in vernalization requirement arises.

Species	Line	Vernalization requirement	Note	References
*B. rapa*	Yellow Sarson (C634)	−	Low level of *BrFLC2* expression in pre-vernalized sample	Li et al., 2009
	Yellow Sarson (L147)	−	Deletion in exon 4 and intron 4 in *BrFLC2*	Wu et al., 2012
	Nou-6 (Chinese cabbage)	++	Low repression rate of *BrFLC1* and *BrFLC5* following vernalization	Kakizaki et al., 2011
	Tsukena No. 2 (Leafy Green/Tsukena)	+++	Low repression rate caused by TE insertion in *BrFLC2* and *BrFLC3*	Kitamoto et al., 2014
*B. oleracea*	Green Comet (broccoli)	−	Frameshift due to 1-bp deletion in exon 4 of *BoFLC2*	Okazaki et al., 2007
	Inbred lines (cauliflower)	−	Frameshift due to 1-bp deletion in exon 4 of *BoFLC2*	Ridge et al., 2015
	E9 (purple sprouting broccoli)	++	High reactivation rate of *BoFLC*2 expression on return to warm condition	Irwin et al., 2016
*B. napus*	Westar etc. (spring type)	−	Loss of BnaFLC.A10 function by TE insertion in the first exon	Yin et al., 2020
			Loss of BnaFLC.A02 function by TE insertion in the exon 7	
	NIL L06 (spring type)	−	Loss of BnaFLC.A02 function by 2,833 bp insertion in the intron 1	Chen et al., 2018
	Zhongshuang 11 etc. (semiwinter type)	+	*BnaFLC.A10* expression level before and following vernalization is lower than that in winter type, which may be due to TE insertion in the promoter region	Yin et al., 2020
	Darmor (European winter type)	++	Higher expression levels of *BnaFLC.A02* before vernalization, lower repression rate following vernalization, and higher reactivation rate on return to warm conditions of *BnaFLC.A02*	Tudor et al., 2020
	Tapidor etc. (European winter type)	++	Low rate of *BnaFLC.A10* repression following vernalization, which may be due to TE insertion in the promoter region	Hou et al., 2012; Yin et al., 2020

*−, low or no vernalization requirement; +, vernalization requirement.*

Loss of FLC function causes markedly early flowering and loss or reduction in the vernalization requirement. However, there is a variation in vernalization requirement among accessions not including early flowering accessions. QTL analysis using a population derived from parental accessions showing different vernalization requirements but both having a vernalization requirement also identified the colocalization of QTLs and the *FLC* gene. Using an F_2_ population derived from a cross between two parental accessions of Chinese cabbage (*B. rapa*) both having a vernalization requirement, QTLs for flowering time colocalized with *BrFLC1* and *BrFLC5* (Kakizaki et al., 2011). The repression rate of *BrFLC1* and *BrFLC5* following cold treatment in the later flowering time accession was lower than in the earlier flowering time accession (Table 2; Kakizaki et al., 2011), suggesting that this different rate of repression of *FLC* expression may be involved in the flowering time difference. It was considered that *BrFLC5* is a pseudogene because of a mutation in the splicing donor site (G to A). However, a functional *BrFLC5* allele has been identified in some accessions and shown to be a weak regulator of flowering time (Xi et al., 2018). We confirmed that the later flowering time accession used in Kakizaki et al. (2011) had a functional *FLC5* allele in spite of sequence basis (G allele), suggesting that colocalization of QTL and *BrFLC5* is due to the difference of BrFLC5 function between parental accessions. QTL analysis was also performed using 194 Chinese cabbage accessions including 40 spring, 37 summer, and 117 autumn types, of which 177 accessions showed a vernalization requirement. The order of increasing vernalization requirement is spring, autumn, and summer accessions. *B. rapa Vernalization Insensitive 3.1* (*BrVIN3.1*) and *BrFLC1* have been identified as sources of variation in bolting time (Su et al., 2018). In *A. thaliana*, *VIN3* is involved in *FLC* silencing during cold treatment and is induced by cold treatment (Kim and Sung, 2013). Five haplotypes of *BrVIN3.1* with sequence variation in the promoter regions have been identified. Three haplotypes of *BrFLC1* with two main groups and one minor group had sequence polymorphism in non-coding regions (Su et al., 2018). These sequence polymorphisms in non-coding regions in *BrVIN3.1* or *BrFLC1* may result in variation in the vernalization requirement in *B. rapa*. In case of the difference in vernalization requirement of *B. oleracea*, a QTL overlapping *BoFLC2* has been identified. There are *cis* polymorphisms in *BoFLC2* that influence the expression dynamics in response to cold treatment especially the reactivation rate on return to warm conditions in purple sprouting broccoli (Table 2; Irwin et al., 2016). In the European winter oilseed rape (*B. napus*), *BnaFLC.A02* (*BrFLC2* ortholog) has been detected as candidate gene giving rise to the QTL. Higher expression levels of *BnaFLC.A02* before cold treatment, lower repression rate following cold treatment, and higher reactivation rate on return to warm conditions of *BnaFLC.A02* were found in the later flowering time accession. The amino acid sequence of BnaFLC.A02 was identical between the earlier and later flowering time accessions. In contrast, there were sequence polymorphisms in non-coding intronic regions between them, which could result in a difference in *FLC* expression and modified vernalization requirement (Table 2; Tudor et al., 2020).

FLC1, FLC2, and FLC3 act as floral repressors in both *B. rapa* and *B. oleracea* (Kim et al., 2007; Itabashi et al., 2019; Takada et al., 2019). It has not been shown whether amino acid sequence differences among functional *FLC* paralogs could lead to differences in the function as floral repressor or variation in vernalization requirement. There is a variation in expression levels among *FLC* paralogs before and following vernalization, and this is also shown in the repression rate of their expression following vernalization (Itabashi et al., 2019; Schiessl et al., 2019; Takada et al., 2019). These results suggest that variation in vernalization requirement is due to the difference in transcriptional repression rate of *FLC* between accessions, and this may be due to sequence polymorphisms in the non-coding regions. Furthermore, the fact that a change in one *FLC* paralog affects vernalization requirement suggests that the *FLC* paralogs may be functioning additively.

## Transposon Insertion Causes Variation of Vernalization Response

Transposable elements (TEs) are DNA sequences that can change their location in the genome, and they can play a role in plant genome evolution (Fujimoto et al., 2012; Hosaka and Kakutani, 2018). In *B. rapa*, TE DNA is methylated, and TEs are epigenetically silenced (Fujimoto et al., 2008b; Sasaki et al., 2011; Takahashi et al.,2018a,b). The variation in the genome structure of orthologous loci between *B. rapa* and *B. oleracea* is due to TE insertions; the *B. oleracea* genome has more TE insertions than the *B. rapa* genome in orthologous genomic regions, which results in a larger genome size in *B. olerace*a than in *B. rapa* (Fujimoto et al., 2006a; Liu et al., 2014). TE insertions can cause loss of protein function or gene expression or generate a new expression pattern (Miura et al., 2001; Fujimoto et al., 2006b, 2008a, 2011; Saze and Kakutani, 2007; Naito et al., 2009; Hosaka and Kakutani, 2018).

A TE insertion has been identified in some alleles of *FLC* in the genus *Brassica*. Long TE insertions in the first intron of *BrFLC2* and *BrFLC3* were detected in the extremely late flowering time accession, Tsukena No. 2 (Kitamoto et al., 2014). In *A. thaliana*, the Landsberg *erecta* (L*er*) accession has a TE in the first intron, and this insertion results in a low level of *FLC* expression (Gazzani et al., 2003). In contrast, insertion of a TE in the first intron of *BrFLC2* or *BrFLC3* in Tsukena No. 2 does not affect the *FLC* expression *per se*. However, weak repression of *BrFLC2* and *BrFLC3* following cold exposure has been detected, suggesting that the TE insertion disrupts the vernalization response (Table 2; Kitamoto et al., 2014). A similar phenomenon has been observed in radish (*Raphanus sativus*); a 1,627-bp insertion in the first intron of *RsFLC2* did not affect the *RsFLC2* expression level before cold treatment but weakened its repression rate during vernalization, resulting in a late-bolting phenotype (Wang et al., 2018). These results suggest that a TE insertion might disrupt a *cis* element of the vernalization response in the first intron or inhibit the recruitment of the POLYCOMB REPRESSIVE COMPLEX 2 (PRC2), which plays a role in epigenetic silencing of *FLC* during vernalization, to the *FLC* locus. In addition to the TE insertion, an insertion/deletion in the second intron of *BoFLC1* may alter gene expression and is associated with variation in flowering time of cabbage varieties (Abuyusuf et al., 2019).

In *B. napus*, there was TE insertion in the promoter region of some *FLC* paralogs, and this insertion affects the vernalization requirement. A 621-bp TE insertion in the upstream region of *BnaFLC.A10* (*BrFLC1* ortholog) was associated with lower rate of *BnaFLC.A10* repression during cold treatment (Table 2; Hou et al., 2012). This 621-bp TE insertion is observed in 73% of winter-type accessions (449 of 619 accessions including Tapidor and Darmor-bzh) and associated with high vernalization requirement (Yin et al., 2020). A 4,422-bp TE insertion is observed in *BnaFLC.A10* of the semiwinter type (Zhongshuang 11, Ningyou 7), and its expression level before and following cold treatment in semiwinter type was lower than in winter types (Table 2). About 57% of semiwinter accessions have this 4,422-bp TE insertion (Yin et al., 2020). A TE insertion results in loss of FLC function in spring-type accessions; a 5,625-bp TE insertion in the first exon of *BnaFLC.A10* (in about 55% of spring-type accessions) and 810-bp TE insertion in the exon 7 of *BnaFLC.A02* (*BrFLC2* ortholog) (about 7%) cause loss of function as does a 2,833-bp insertion in the first intron of *BnaFLC.A02* (about 18%) (Table 2; Chen et al., 2018; Yin et al., 2020).

These data indicate that TE insertions play an important role in the determination of flowering time and vernalization requirement in the genus *Brassica*.

## Histone Modification Triggers Epigenetic Silencing of the *FLC* Following Vernalization

Histone modification is an epigenetic modification that plays a role in the regulation of gene expression (Pfluger and Wagner, 2007; Richards, 2011; Fujimoto et al., 2012). Genome-wide histone modification states have been examined by chromatin immunoprecipitation sequencing (ChIP-seq) in many plant species. In *B. rapa*, di-methylation of histone H3 lysine 9 (H3K9me2) and trimethylation of histone H3 lysine 27 (H3K27me3) have been mapped genome wide (Takahashi et al., 2018a; Akter et al., 2019; Payá-Milans et al., 2019). In *B. rapa*, H3K9me2 was overrepresented in TEs, and there were low number of genes having H3K9me2 marks (Takahashi et al., 2018a). About 30% of genes had H3K27me3 marks, and H3K27me3 marks were associated with low level of gene expression and high level of tissue-specific gene expression (Akter et al., 2019; Payá-Milans et al., 2019). H3K27me3 modification is catalyzed by the PRC2, and in *B. rapa*, a mutant of the *CURLY LEAF* (*CLF*) gene, which is the enzymatic core of PRC2, showed decreased H3K27me3 level (Payá-Milans et al., 2019), suggesting that PRC2 plays a role in H3K27me3 in *B. rapa* as in other plant species.

**FIGURE 1:**
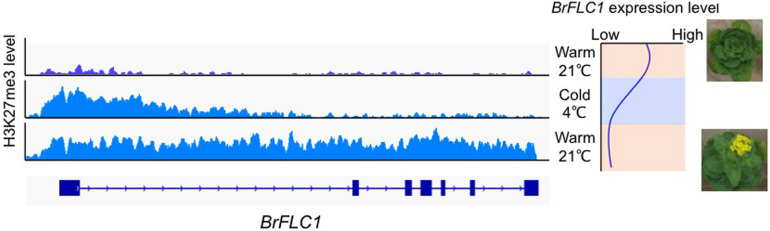
H3K27me3 pattern during vernalization in *BrFLC1*. Left panel shows the pattern of accumulation of H3K27me3 marks during vernalization. Right panel shows the expression pattern of *BrFLC1* during vernalization. *BrFLC2*, *BrFLC3*, and *BrFLC5* showed similar H3K27me3 pattern to *BrFLC1* (Akter et al., 2019).

In *A. thaliana*, repression of *FLC* expression during vernalization is associated with switching of chromatin states from enrichment of active histone marks (H3K4me3, H3K36me3) to repressive histone marks (H3K27me3) at the *FLC* locus (Berry and Dean, 2015). During prolonged cold treatment, there is accumulation of H3K27me3 within the nucleation region, which consists of a DNA region associated with one or two nucleosomes and harboring the first exon and the start of first intron. H3K27me3 spreads over the entire region of *FLC* when temperatures are warm (Xi et al., 2020). A *cis* DNA element in the first intron of *FLC* [termed the RY element (CATGCA)] that is essential for *FLC* repression during vernalization and the maintenance of *FLC* silencing when temperatures are warm has been identified (Qüesta et al., 2016; Yuan et al., 2016). In *B. rapa*, H3K4me3 accumulation was found in the nucleation region of all four *BrFLC* paralogs before exposure to cold (Kawanabe et al., 2016). During vernalization, accumulation of H3K27me3 within the nucleation region of all four *BrFLC* paralogs with decreased expression was observed (Akter et al., 2020; Figure 1). After return to warm conditions, H3K27me3 accumulation spreads across the four *BrFLC* paralogs maintaining silencing (Kawanabe et al., 2016; Akter et al., 2019; Figure 1). The position of the nucleation region is conserved between *A. thaliana* and *B. rapa*, but there is less conservation of first intron sequences (Figure 1). Two RY elements are conserved in four *BrFLC* paralogs except in *BrFLC1* where there is a mutation in one of the two RY elements. Like in *A. thaliana*, PRC2 could be recruited to the nucleation regions of the four *BrFLC* paralogs (Akter et al., 2019).

The genome-wide level of H3K27me3 in *B. rapa* was compared between non-vernalized, 4 weeks vernalized, and sample upon return to warm temperature after 4 weeks vernalization. Between the non-vernalized and 4 weeks vernalized samples, 6,814 genes showed a difference in H3K27me3 level, and 99% of genes showed decreased H3K27me3 following vernalization. Between the non-vernalized and the sample upon return to warm temperature after 4 weeks vernalization, 189 genes showed a difference in H3K27me3 level, and 85% of genes showed increased H3K27me3 following vernalization. In *A. thaliana*, 380 genes showed a difference in H3K27me3 level between non-vernalized and sample upon return to warm temperature following 4 weeks vernalization, and only the *FLC* gene showed increased H3K27me3 levels following vernalization (Akter et al., 2019). A trend toward increased levels of H3K27me3 following vernalization in *A. thaliana* has been reported (Xi et al., 2020). A limited number of genes such as *B. rapa MADS AFFECTING FLOWERING* (*BrMAF*) genes, which are related to *FLC*, showed a similar H3K27me3 accumulation pattern; H3K27me3 accumulated in a specific region during vernalization and then spread upon returning to warm temperatures after vernalization (Akter et al., 2019). At the transcriptional level, some genes showed a similar expression pattern to *FLC* during cold treatment in *A. thaliana* (Xi et al., 2020). Some genes showed a change in H3K27me3 accumulation following vernalization, but only a limited number of genes showed an H3K27me3 modification pattern similar to that of *FLC*, where vernalization leads to H3K27me3 accumulation in the specific regions (nucleation region) and then spread throughout the gene when temperature is returned (Akter et al., 2019).

## Are Long Non-Coding Rnas Vernalization Players?

**FIGURE 2:**
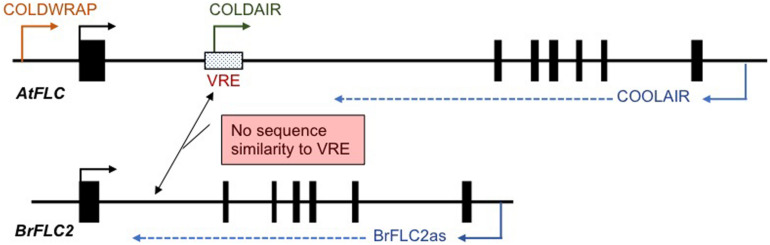
Schematic view of the position of cold-induced non-coding RNAs in *A. thaliana* and *B. rapa*. There is no sequence similarity between the putative vernalization response element (VRE) in *A. thaliana* and the first intron of four *BrFLC* paralogs. Only *BrFLC2* has antisense transcripts, BrFLC2as.

In *A. thaliana*, three cold-induced non-coding RNAs (COOLAIR, COLD ASSISTED INTRONIC NON-CODING RNA (COLDAIR), and COLDWRAP) have been identified. COOLAIR is transcribed in the antisense direction, and COLDAIR and COLDWRAP are transcribed in the sense direction relative to *FLC* messenger RNA (mRNA) transcription. COOLAIR transcripts start from downstream of the poly-A site to the promoter region of *FLC* (Figure 2; Swiezewski et al., 2009). COLDAIR is derived from the first intron and COLDWRAP from the promoter region (starts at 225 bp upstream from transcription start site) of *FLC* (Figure 2; Heo and Sung, 2011; Kim and Sung, 2017). These non-coding RNAs are involved in *FLC* silencing and maintenance of the repressed condition (Swiezewski et al., 2009; Heo and Sung, 2011; Kim and Sung, 2017; Whittaker and Dean, 2017). COOLAIR-like transcripts have been identified in five species of Brassicaceae, *Arabidopsis lyrata*, *Arabis alpina*, *Capsella rubella*, *Eutrema salsugineum*, and *B. rapa*. Although there is low conservation of COOLAIR-like sequences between species, secondary structures show similarity, suggesting that the functional role, the regulation of *FLC* expression during vernalization, may be conserved in Brassicaceae species (Hawkes et al., 2016).

In *B. rapa*, COOLAIR-like transcripts have been identified only at the *BrFLC2* locus and are termed BrFLC2as, while no COOLAIR-like transcript was detected from the other three *BrFLC* loci (Figure 2; Li et al., 2016; Shea et al., 2019). COOLAIR has two transcripts, proximal site (class I, ∼400 nt) and distal site (class II, ∼750 nt), and BrFLC2as also has two different length transcripts, class I (short, 357, 425, and 510 nt) and class II (long, 665 and 767nt) (Li et al., 2016). BrFLC2as was highly induced by short-term cold treatment, and class II BrFLC2as was at a higher level than class I BrFLC2as (Li et al., 2016; Shea et al., 2019). Similarity of secondary structure and the characteristic of being induced by short-term cold treatment in both BrFLC2as and COOLAIR suggests a functional similarity (Li et al., 2016). However, the other three *BrFLC* paralogs also show decreased expression during vernalization in spite of no cold-induced COOLAIR-like transcripts being detected (Li et al., 2016; Shea et al., 2019). BrFLC2as may be involved in the silencing of the other three *BrFLC* paralogs. Overexpression of the BrFLC2as resulted in reduced *BrFLC1*, *BrFLC2*, and *BrFLC3* expression leading to an early flowering phenotype, suggesting that BrFLC2as may be involved not only in the repression of *BrFLC2* but also in the repression of the *BrFLC1* and *BrFLC3* genes (Li et al., 2016). However, this experiment does not rule out the possibility that repression of the three *BrFLC* genes is due to posttranscriptional gene silencing because the introduced BrFLC2as sequence includes the exon 1 region of *BrFLC2* and may silence all three genes (Li et al., 2016). Furthermore, there is a question whether BrFLC2as is able to function in *trans* because COOLAIR functions in *cis* (Csorba et al., 2014). Further study will be needed to elucidate the role of BrFLC2as in the vernalization mechanism in *B. rapa*.

In *A. thaliana*, COLDAIR and COLDWRAP play a role in recruitment of PRC2 to the *FLC* locus (Kim and Sung, 2017). However, neither COLDAIR nor COLDWRAP-like transcripts nor other cold-induced transcripts from the promoter or first intron have been identified in the four *FLC* paralogs of *B. rapa* (Li et al., 2016; Shea et al., 2019). However, accumulation of H3K27me3 was confirmed in all four *BrFLC* paralogs (Akter et al., 2019), leading to the question of how PRC2 is recruited to the *BrFLC* loci during vernalization.

Cold-induced natural antisense transcripts (NATs) were also identified in two *BrMAF* genes in *B. rapa*. In *B. rapa*, there are five *BrMAF* genes, and three of these five *BrMAF* genes were repressed following vernalization (Akter et al., 2019). Accumulation of H3K27me3 during vernalization also has been shown in these three *BrMAF* genes (Akter et al., 2019). In addition, MAF1 and MAF2 isolated from Pak-choi (*B. rapa* var. *chinensis*), termed BcMAF1 and BcMAF2, respectively, function as floral repressors (Huang et al., 2018, 2019). These cold-inducible NATs derived from *BrMAF* loci may have a similar function to COOLAIR. In *A. thaliana*, *MAF4* has an overlapped NAT termed MAS. *MAF4* is induced early in cold treatment, and MAS is coordinately expressed during the cold treatment (Zhao et al., 2018). Repression or silencing of *MAF4* expression did not affect MAS expression, while repression of MAS expression reduced *MAF4* induction. These results suggest that MAS plays a positive role in *MAF4* expression. In *B. rapa*, coordinate expression of mRNA and the NAT pair following vernalization was observed in some genes but not in *BrMAF* genes (Shea et al., 2019). This coordinate expression in mRNA and NAT pairs may have a role in vernalization or the cold response.

## Perspective

Whole genome triplication results in multiple copies of *FLC* paralogs in the genus *Brassica*. FLC1, FLC2, and FLC3 act as floral repressors in both *B. rapa* and *B. oleracea* (Kim et al., 2007; Itabashi et al., 2019; Takada et al., 2019), suggesting that all three *FLC* paralogs need to be considered to understand the vernalization mechanism. There is a difference in transcriptional level before vernalization among three *FLC* paralogs in both *B. rapa* and *B. oleracea*, and between accessions within species, there are differences in which *FLC* paralog shows the highest expression (Itabashi et al., 2019; Takada et al., 2019). A similar situation is observed in *B. napus*, which has nine *FLC* paralogs (Schiessl et al., 2019). Different expression levels before vernalization between paralogs could be due to sequence polymorphisms in the promoter regions. In addition, sequence polymorphisms in the promoter region could also be involved in the difference in expression levels in each *FLC* between accessions. The correlation between the total level of *FLC* expression before vernalization and the vernalization requirement suggests that the level of *FLC* expression before vernalization is associated with the diversity of vernalization requirement in *B. rapa* (Takada et al., 2019). We need to identify the region or *cis*-element controlling *FLC* expression level to understand the variation in vernalization requirement between accessions.

There is also a variation in the rate of repression of *FLC* expression among paralogs during vernalization with a difference between accessions as to which *FLC* paralog shows a low repression rate. A low repression rate of *FLC* genes is an important factor in generating a vernalization requirement, and in this review, some examples are shown such as a TE insertion in the first intron or a TE insertion in the promoter region, which affect this requirement (Gazzani et al., 2003; Hou et al., 2012; Kitamoto et al., 2014; Yin et al., 2020). There is a need to clarify the complexity that arises from multiple *FLC* paralogs. Furthermore, non-coding RNAs, which can recruit PRC2 to *FLC* loci, have not been identified, and a vernalization responsive element (VRE) in the genus *Brassica* has not been identified. Identification of a VRE may clarify the association between VRE sequence polymorphism and variation in repression rate following vernalization between paralogs or between accessions within species.

## Author Contributions

TK, KO, ED, and RF conceptualized the manuscript. AA, EI, ED, and RF wrote the manuscript. TK, KO, ED, and RF edited the manuscript. All authors contributed to the article and approved the submitted version.

## Conflict of Interest

The authors declare that the research was conducted in the absence of any commercial or financial relationships that could be construed as a potential conflict of interest.
